# Patterns of Change in Nucleotide Diversity Over Gene Length

**DOI:** 10.1093/gbe/evae078

**Published:** 2024-04-12

**Authors:** Farhan Ali

**Affiliations:** Biodesign Center for Mechanisms of Evolution, Arizona State University, Tempe, AZ 85281, USA

**Keywords:** synonymous polymorphism, effective population size, purifying selection, minimum doubling time, recombination, phylogenetic comparison

## Abstract

Nucleotide diversity at a site is influenced by the relative strengths of neutral and selective population genetic processes. Therefore, attempts to estimate Effective population size based on the diversity of synonymous sites demand a better understanding of their selective constraints. The nucleotide diversity of a gene was previously found to correlate with its length. In this work, I measure nucleotide diversity at synonymous sites and uncover a pattern of low diversity towards the translation initiation site of a gene. The degree of reduction in diversity at the translation initiation site and the length of this region of reduced diversity can be quantified as “Effect Size” and “Effect Length” respectively, using parameters of an asymptotic regression model. Estimates of Effect Length across bacteria covaried with recombination rates as well as with a multitude of translation-associated traits such as the avoidance of mRNA secondary structure around translation initiation site, the number of rRNAs, and relative codon usage of ribosomal genes. Evolutionary simulations under purifying selection reproduce the observed patterns and diversity–length correlation and highlight that selective constraints on the 5′-region of a gene may be more extensive than previously believed. These results have implications for the estimation of effective population size, and relative mutation rates, and for genome scans of genes under positive selection based on “silent-site” diversity.

**Significance**

Synonymous sites towards the 5′-end of genes show less variation than the average due to strong purifying selection on translation initiation rates in bacteria. However, the extent to which this selection shapes the patterns of variation across the genome, and across species, has not been investigated. The present study finds that the effect of this selection is strong enough to result in an unexpected correlation between a gene's length and its degree of per-site polymorphism and that the distribution of these effects correlates with the variation in maximal growth rates of different species. Future studies estimating effective population size and employing genome-wide tests of positive selection should benefit from recognizing these selective constraints on variation at “silent” sites.

## Introduction

Populations in nature are known to be genetically polymorphic. The extent of this polymorphism varies across species as well as across loci within a genome (Frankham 1996; Kaas et al. 2012; Lee and Mitchell-Olds 2012; Martincorena et al. 2012; Cornejo et al. 2015; Echave et al. 2016; Figuet et al. 2016; Andreani et al. 2017). Understanding the basis of this variation and the relative contribution of selective and neutral processes remains a central problem in evolutionary biology (Zhang and Yang 2015).

The efficacy of natural selection in shaping patterns of molecular variation depends on the strength of random genetic drift, measured as the inverse of effective population size (1/*N*_e_). In the absence of selection, the nucleotide heterozygosity of a bacterial population (π) is expected to equilibrate at 2 *N*_e_  *μ* where *μ* is the number of mutations per base-pair per generation (Maruyama and Kimura 1980). Thus, *N*_e_ can be estimated given mutation rates and a class of nucleotide sites that evolve nearly neutrally. Conventionally, 4-fold degenerate sites in the protein-coding genes are used for this purpose (Lynch and Conery 2003; Cutter 2013). However, a variety of selective mechanisms have been uncovered that can shape diversity at synonymous sites (Schattner and Diekhans 2006; Park et al. 2013; Bailey et al. 2021). Some of these mechanisms may be localized to specific regions or sites (Eyre-Walker and Bulmer 1993; Akashi 1994). Hence, to improve our estimates of *N*_e_, and thereby our understanding of the evolution of specific traits, we need to distinguish synonymous sites evolving nearly neutrally from those under stronger constraints.

In our study on the variability of transcription factors and their target genes in *E. coli*, we found nucleotide diversity of genes to be positively correlated to their length and this effect was stronger for synonymous sites in comparison to nonsynonymous sites (Ali and Seshasayee 2020). Nucleotide diversity at a locus is calculated as the average pairwise difference per site among sequences in a population and so, is not expected to show any correlation with gene length. Since nucleotide diversity is also a fundamental measure of variation in population genetics (Lynch and Crease 1990; Chakravarti 1999), and provides a basis for common tests of neutral evolution (Tajima 1989; McDonald and Kreitman 1991), any factor that shapes its distribution within or across species must be clearly understood.

Variation in synonymous diversity across genes could be a reflection of varying degrees of selection on synonymous codons. Selection on codon usage has been observed to be correlated to gene length in *Escherichia coli* (Eyre-Walker 1996a) and *Drosophila* (Comeron et al. 1999). Independently, a pattern of low synonymous substitution rates at both ends of a gene has been observed in many organisms (Eyre-Walker and Bulmer 1993; Molina and Nimwegen 2008; Johri et al. 2017; Lynch et al. 2017), which is likely due to selection against mRNA secondary structure around ribosome-binding sites and initiation codons for efficient translation initiation (Gu et al. 2010). Consequently, this is believed to operate within the first 50 bases of the translation initiation site (TIS) (Molina and Nimwegen 2008), and thus, would seem unlikely to be an explanation for the observed correlation of synonymous diversity with gene length. However, it could be that the region over which this selection operates extends beyond the first 50 sites and varies in length across species. We can resolve this issue by quantifying these patterns of synonymous polymorphism in a more systematic manner than attempted previously. More generally, the strength of selection acting on translational efficiency in a bacterial species is expected to correlate to its growth rate. Thus, if translational selection drives the gene-length dependence of synonymous diversity, we may expect these patterns to be more evident in species with higher growth rates. However, any correlation with growth rate does not imply that the selection shaping patterns of synonymous diversity is indeed translational.

In this paper, I first develop a method to quantify the average pattern of synonymous diversity over the length of *E. coli* genes. Then, I apply this method to multiple bacterial species in order to study how the strength of diversity–length correlation varies across species. After controlling for statistical correlates, I test the effect of recombination rates and several metrics of enhanced translation rates on the distribution of observed patterns of synonymous polymorphism across species. Finally, I test through evolutionary simulations whether purifying selection is sufficient to generate the observed patterns of diversity and its correlation with gene length.

## Results

### Diversity–length Correlation Reflects Reduced Polymorphism Toward Gene Starts

**Fig. 1.**
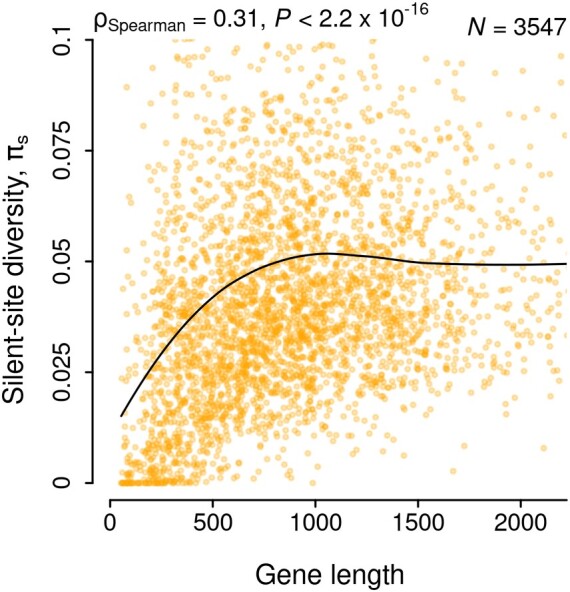
Correlation between nucleotide diversity and gene length. Nucleotide diversity was estimated from silent sites (4-fold degenerate) of 3547 genes present in at least 75% of the 96 *E. coli* genomes. *X* and *Y* axes are limited to their respective upper 95th percentile for visualization. The black line shows the LOESS curve with a span of 0.75 and highlights the broad trend of change in synonymous diversity with gene length.

We have seen previously that nucleotide diversity is positively correlated with gene length in *Escherichia coli* (Ali and Seshasayee 2020). The strength of this correlation was stronger for synonymous sites, suggesting that the process driving this length dependence was directly acting on the nucleotide sequence. In this study, I estimate diversity from 4-fold degenerate sites to eliminate the effect of any selection acting on amino-acid sequences. For a collection of 96 *E. coli* genomes, selected as described in Methods, the positive correlation between gene length and silent-site diversity was evident, with a distinctly nonlinear, saturating trend (Fig. 1). This could result from an underlying pattern of site-to-site variation in nucleotide diversity such that shorter genes have a smaller proportion of high-diversity sites. Such patterns of reduced variation near translation initiation sites (TIS) have been observed previously (Eyre-Walker and Bulmer 1993; Molina and Nimwegen 2008; Johri et al. 2017; Lynch et al. 2017). The primary question here is whether these patterns can explain the observed diversity–length correlation. Since the patterns over individual genes are highly variable (supplementary fig. S1, Supplementary Material online), I instead focus on the trend of average nucleotide diversity over sites, quantify the magnitude and reach of this effect, and compare patterns across species to understand the basis of the observed correlation between gene length and nucleotide diversity.

**Fig. 2.**
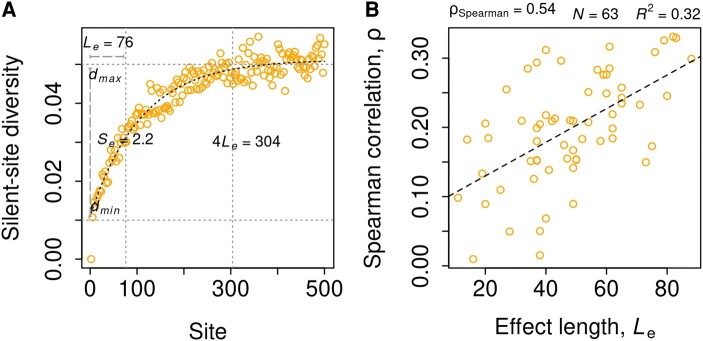
Patterns of reduced diversity toward the start of bacterial genes. A) ASR fit to mean nucleotide diversity of 4-fold degenerate sites averaged over *E. coli* genes. *L*_e_ and *S*_e_ mark the estimates of Effect Length and Size respectively, quantifying the magnitude and extent of the observed reduction, respectively. B) Linear regression of the strength of correlation between gene length and silent-site diversity on estimates of *L*_e_ for 63 bacterial species with *S*_e_ > 0 and *ρ* > 0.

As an asymptotic trend in the nucleotide diversity of a site averaged over genes is apparent from Fig. 2A, a natural choice of fit for diversity-site profiles would be the asymptotic regression (ASR) model. Such a negative exponential equation can be used to quantify the magnitude and extent of this effect on diversity, which can be compared across samples of strains or species. Here onward, “Effect Length *L*_e_” is defined as the number of silent sites from the translation starts at which the estimated average diversity is halfway to saturation, and “Effect Size *S*_e_” is defined as the log-2 fold difference between the maximum and minimum estimated mean diversity. Effect Length in *E. coli* was estimated as equal to 76 (Fig. 2A). The point of saturation can be conservatively approximated as 4 *L*_e_ = 304. The estimated Effect Size of 2.2 captures the nearly 5-fold decrease in diversity at the start site relative to its expected value at saturation.

**Fig. 3.**
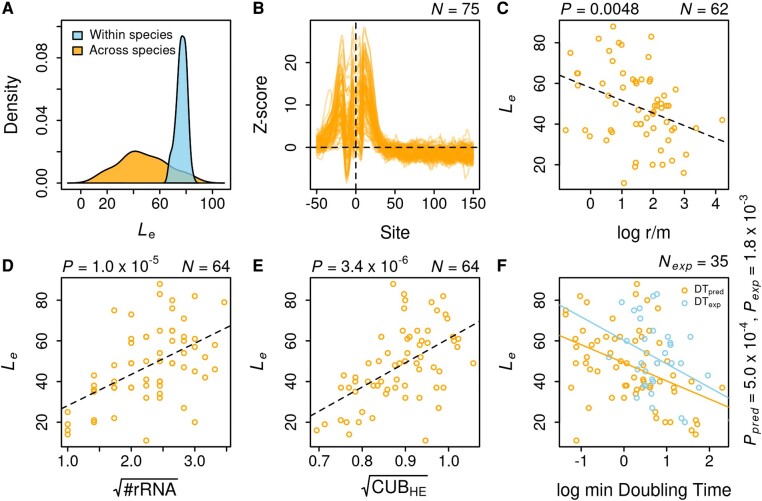
Effect Length and its predictors across species. A) Distribution of Effect Length within and across species. Within-species distribution is represented by a random sample, of the same size as the number of species, drawn from a Normal distribution with mean and standard deviation equal to the estimate of Effect Length in *E. coli* and its standard error, respectively. Across-species distribution is more dispersed with its lower range of values well beyond the distribution of Effect Length in *E. coli*. B) Selection to avoid mRNA secondary structure around TIS. Z-score profiles for the probability of a base being unpaired in an mRNA secondary structure were drawn for 75 species. Z-scores for different species show considerable variation in their maximum values but attain baseline within a narrow range of positions. C) Variation in Effect Length can be partially explained by species-specific recombination rate. The recombination rate was measured using Mcorr as a log-ratio of the rate of recombination to mutation D) & E). Effect Length was strongly correlated with rRNA count and relative codon usage bias of highly expressed genes, respectively. Both predictors were squared-root transformed based on their Box–Cox *λ*. F) Effect Length is negatively correlated with predicted as well as experimentally observed minimum doubling times. Doubling times are in hours. Natural logarithm of doubling times was taken for appropriate Box–Cox transformation. All mentioned *P*-values are for simple linear regression of Effect Length on a given predictor.

Using 75 species with at least 30 genomes, the ASR model described above could be fit to the mean nucleotide diversity profile of 69 species, 65 of which had a positive Effect Size. Species without a fit or with a negative Effect Size have considerably lower diversity (π_q = 0.95_ = 5.06 × 10^−3^) compared to the others (π_q = 0.05_ = 6.12 × 10^−3^). Thus, over 85% of the analyzed species showed clear signs of reduced polymorphism towards the 5′-end of genes. The estimates of Effect Length ranged from 11 in *Streptococcus thermophilus* to 88 in *Acinetobacter baumannii*. The variability in Effect Length estimates across species was greater than that of the Effect Size, both in terms of their observed coefficients of variation, 0.39 v/s 0.32, and as the ratios of observed standard deviation to the corresponding standard error in *E. coli*, 4.72 v/s 3.94 (Fig. 3A). Estimates of *L*_e_ and *S*_e_ across species were correlated to each other (*ρ*_Spearman_ = 0.46), as well as to the strength of the correlation between gene length and nucleotide diversity [(*L*_e_) = 0.54, (*S*_e_) = 0.45]. Multiple linear regression of this correlation coefficient (*ρ*_l_) on *L*_e_ and *S*_e_ found the effect of *L*_e_ to be much stronger than that of *S*_e_ [*P*(*L*_e_) = 0.0004, *P*(*S*_e_) = 0.0462]. A simple linear regression with *L*_e_ alone explained nearly one-third of the variation in the degree of gene-length dependence of nucleotide diversity (Fig. 2B). In conjunction with the Effect Size and the estimate of maximum diversity (*d*_max_), nearly 40% of the variance could be explained (*R*^2^ = 0.39). The rest of this study is focused on understanding the factors that shape the distribution of Effect Length across species.

### Effect Length Correlates With Selection Against mRNA Secondary Structure and the Rate of Recombination

The observed distribution of Effect Lengths across species could be biased by confounding variables. Before proceeding with understanding the effect of biological factors, I tested for the correlation of Effect Length with potential statistical confounders, such as the number of strains, number of genes, average gene length, mean nucleotide diversity, and GC content (supplementary Text S1, Supplementary Material online). Only the number of strains had a significant correlation with Effect Length and was controlled for in further analyses (supplementary table S1, Supplementary Material online).

The most common explanation for reduced polymorphism at 5′-ends of protein-coding genes seems to be the selection to avoid mRNA secondary structure formation around TIS (Eyre-Walker and Bulmer 1993; Rocha et al. 1999; Molina and Nimwegen 2008). However, this selection is believed to operate within the first 50 bp of CDS, and consequently, attempts to quantify the magnitude and extent of this effect have focused only on this limited region. To test if the selection of mRNA secondary structure can explain the variation in *L*_e_ across species, I used RNA secondary structure prediction over a larger region i.e. 100 bases upstream to 200 bases downstream of TIS, and estimated the average secondary-structure free length (*L*_ss_) for each species. More specifically, I calculated the probability of each base being unpaired across an ensemble of possible RNA secondary structures over the specified region of every gene in a genome. I converted raw probabilities to Z-scores following Molina's approach (Molina and Nimwegen 2008), such that the Z-score measures the tendency of a base to be unpaired relative to the bases at the ends of the segment. I defined Effect Length (*L*_ss_) and Effect Size (*S*_ss_) in this context as the first site at which Z-score drops to 0 and as the maximum Z-score, respectively. *L*_ss_ for 75 species ranged from 23 to 114, with limited dispersion, as indicated by its inter-quartile range (IQR = Q_3_–Q_1_) of 9, relative to that of *L*_e_ with IQR = 24 (Fig. 3B). *L*_e_ has a negligible correlation with *L*_ss_ (*ρ*_Spearman_ = −0.08, *P* = 0.51, *N* = 64) but a significant correlation with *S*_ss_ (*ρ* = 0.27, *P* = 0.03). Even when accounting for the number of strains in a multiple regression model, the effect of *S*_ss_ on *L*_e_ remains significant [*P*(*S*_ss_) = 0.0187, *P*(*N*_strains_) = 0.0048]. Hence, while the length over which the selection to avoid mRNA secondary structure operates is relatively more uniform, patterns of low diversity near TIS do exhibit a weak dependence on the strength of this effect across species.

Even if the strength of selection was uniform, Effect Length could still differ among species due to the varying extent of linkage disequilibrium. If recombination is infrequent, then even the neutral sites linked to loci under purifying selection will have reduced polymorphism (Nordborg et al. 1996). I tested this idea by estimating the ratios of recombination-to-mutation rate per nucleotide site using Mcorr [Methods] (Lin and Kussell 2019). Mcorr uses a coalescent-based approach to estimate the recombinational and mutational divergence of a bacterial population given a sample. Out of 64 species with Effect Length, r/m of 2 species had extreme values *viz.*, *Bordetella parapertussis* (<10^−3^) and *Wolbachia* (>10^3^), leaving 62 species. Linear regression along with the number of strains identified a significantly negative effect of r/m on *L*_e_ (*P* = 0.0097) (Fig. 3C). The model had a strong phylogenetic signal (Pagel's *λ* = 0.7), even accounting for which didn’t eliminate the effect of recombination rate on *L*_e_ (*P* = 0.0152). Therefore, low levels of recombination may extend the regions of reduced diversity around TIS beyond the short length over which the selection to avoid mRNA secondary structure operates.

### Effect Length Reflects Growth Phenotype of a Species

The avoidance of the mRNA secondary structure around the translation start site, as discussed above, is a mechanism to improve translation initiation rates through greater accessibility of ribosomes to their binding sites (Smit and Duin 1990). All else being equal, a more efficient translation should lead to faster growth of the species. Since a greater magnitude of this selection leads to a longer Effect Length, one might expect Effect Length to correlate with improved growth phenotypes. However, the growth characteristics of the majority of bacteria are unknown as they remain unculturable, and even for those that can be cultured, their growth potential might not be realized in laboratory conditions. In the absence of experimentally verified growth rates for many species, I used genome-based predictions of minimum doubling times to test this idea. More specifically, I used the gRodon program that employs relative codon usage bias of highly expressed genes (CUB_HE_) and additional metrics to predict the minimum doubling time for a given genome (Weissman et al. 2021). Additionally, I used the average rRNA count (*N*_rrn_) for each species as the number of rRNAs is known to correlate with faster growth (Vieira-Silva and Rocha 2010). All of the 4 variables *viz.*, CUB_HE_, *N*_rrn_, *L*_e_, and predicted minimum doubling times (DT_pred_) were correlated to each other (Fig. 3D-F, Table 1). *L*_e_ was more strongly correlated with CUB_HE_ and *N*_rrn_ than with DT_pred_ itself. Considering collinearity among CUB_HE_, *N*_rrn_ and DT_pred_, Variance Inflation Factor (VIF) of CUB_HE_ and DT_pred_ was comparable to each other and greater than that of *N*_rrn_ (supplementary table S2, Supplementary Material online). Since DT_pred_ is additionally a composite measure based on other genome characteristics, I chose to include CUB_HE_ in the following regression analysis instead of DT_pred_. Multiple linear regression of *L*_e_ on *N*_rrn_ and CUB_HE_, along with *N*_strains_, found all three predictors to be relevant (supplementary table S2, Supplementary Material online). The residuals of this model showed a weak phylogenetic signal [Pagel’s *λ* = 0.1 (−0.19, 0.4: 95%CI)], even accounting for which did not change the above results.

**Table 1:** Spearman correlation among Effect Length and its covariates

	*S*_ss_	ln r/m	√N_rrn_	√CUB_HE_
ln r/m	0.04	…	…	…
√N_rrn_	0.25	−0.04	…	…
√CUB_HE_	0.38	−0.27	0.58	…
*L*_e_	0.26	−0.35	0.47	0.51

The application of genomic attributes to test for a relationship between Effect Length and maximum growth rate is not entirely satisfactory since it is possible that the Effect Length is associated with other aspects of these genomic traits that are unrelated to their effect on growth rates. Any relationship between Effect Length and growth rates would be far more convincing if corroborated with experimental data. Minimum doubling times determined through cell cultures are indeed available for at least half of the species used in this study. However, one of the main issues in using experimental growth rates is the variation caused due to different laboratory growth conditions, in particular, temperature. In the most recent collection of experimental rates, a correction for differences in temperature was applied by normalizing all growth rates to 20 °C assuming Q_10_ = 2.5 (Lynch et al. 2022). Such normalized growth rates were available for 31 of the species used in this study. For another four species, growth rates were taken from a previous collection (Vieira-Silva and Rocha 2010) and normalized for temperature using the same approach as above. Finally, with these experimentally determined, minimum doubling times (h) of 35 species, I tested for a relationship between Effect Length and maximum growth rates. Effect Length was indeed correlated with minimum doubling times (*ρ*_Spearman_ = −0.5, *P* = 0.004, *R*^2^ = 0.24), suggesting that Effect Lengths across species may be shaped by their growth characteristics.

### Growth-associated Genetic Traits and Recombination Rates are Significant Predictors of Effect Length Across Species

Having studied the association of individual factors with Effect Length and found a set of key correlates, the final step was to identify the most effective predictors and their independent effects. Multiple linear regression of *L*_e_ on *S*_ss_, r/m, *N*_rrn_, CUB_HE_ and *N*_strains_ using 62 species identified rRNA count and recombination rate as the top two significant predictors followed by the number of strains. I compared the full model against simpler models using a backward elimination procedure with a 10-fold cross-validation repeated 100 times. The full model turned out to be the best model as judged by its minimum prediction RMSE (RMSE = 14.38, *R*^2^ = 0.43), with rRNA count and recombination rate as the two most effective predictors (Table 2).

**Table 2:** Regression models of Effect Length on different subsets of predictors

	*S*_ss_	ln r/m	√N_rrn_	√CUB_HE_	N_strain_	RMSE	*R* ^2^	MAE
1	−	−	+	−	−	16.44	0.31	13.26
2	−	+	+	−	−	16.12	0.32	12.90
3	−	+	+	−	+	15.88	0.33	12.71
4	−	+	+	+	+	14.50	0.43	11.56
5	+	+	+	+	+	14.38	0.43	11.37

The models with minimum test Root Mean Squared Error (RMSE) in each category of the number of predictors have been listed. Plus(+) signs mark the variables corresponding to each model. The last column lists the Mean Absolute Error of each model (MAE).

The phylogenetic signal in this model was weak enough to be ignored in favor of a simpler model [Pagel’s *λ* = 0.1 (−0.31:0.52, 95% CI)]. However, this lack of phylogenetic signal is expected with a large number of parameters and few observations, and thus cannot be taken as evidence of phylogenetic independence of the distribution of Effect Length as governed by the above factors. To test for true independence, I split the dataset by the two major phylogenetic groups i.e. Terrabacteria (*N* = 24) and Gracilicutes (*N* = 38) (supplementary fig. S2, Supplementary Material online). Gram-positive and Gram-negative bacteria belong to the groups Terrabacteria and Gracilicutes, respectively. The full models trained on one group and tested on the other performed poorly (*R*^2^ ∼ 0.07), which highlights the phylogenetic dependence of these relationships. While these individual models are useful for demonstrating the phylogenetic dependence of Effect Length on its covariates, they lack the power to reveal all relevant relationships in this limited dataset. In the absence of extensive population genomic data presently, all of the above predictors may be considered relevant to the full distribution of Effect Length across species.

### Extensive Purifying Selection Towards the 5′-end of Genes Can Explain the Diversity–length Correlation

The above correlative analysis suggests that the variation in the strength of purifying selection at the TIS along with recombination rate variation may be sufficient to explain the distribution of Effect Length. To test whether this is true, I used forward evolutionary simulations of bacterial populations under the Wright–Fisher (WF) model. A WF model assumes a single population of constant size, no subdivision, and nonoverlapping generations of reproduction. Deleterious mutations occurred only in the initial regions of genes with every site within the region under the same strength of selection and the rest of the sites were completely neutral. I sampled the values of mutation and recombination rate, and the strength and length of the selection region from their observed distribution across species in this study, setting *N* = 5,000. Simulations followed the evolution of the same randomly generated 50 Kb chromosomal segment over 20,000 generations under different combinations of the above parameters. Further details about the simulations can be found in the Methods section.

**Fig. 4.**
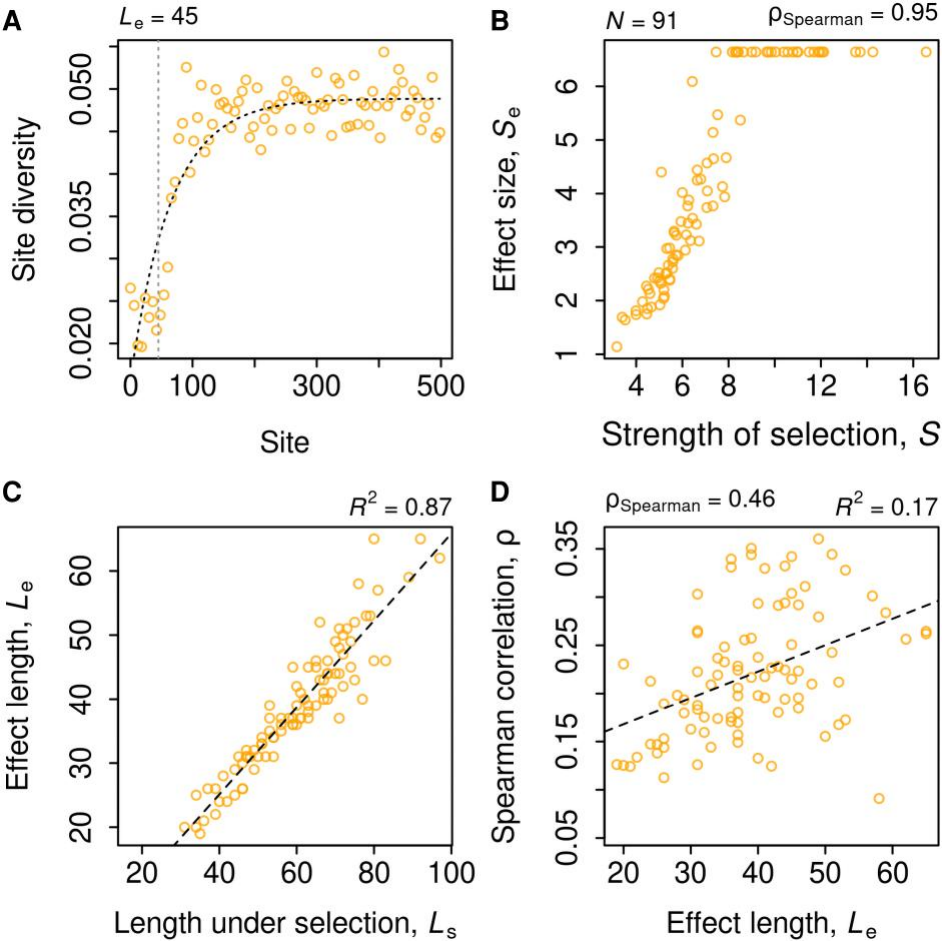
Evolutionary simulations of the patterns of reduced diversity under purifying selection. A) An example of ASR fit to the average gene position diversity of a simulated species. B) Correlation of Effect Size *S*_e_ with simulation parameter—the relative strength of negative selection (*S* = |2 *N*_e_  *s*|)—for 91 simulated species with *S*_e_ > 0. C) Linear regression of Effect Length *L*_e_ on the simulation parameter—the length of the region under selection (*L*_s_). D) *L*_e_ as a predictor of diversity–length correlation.

The mean site diversities of the simulated species showed a similar pattern over gene length as in the real species (Fig. 4A). The ASR could be applied to 91 of the 100 simulated species. Effect Size and Effect Length varied over the range of 1.14 to 6.64 & 19 to 65 respectively. Estimates of Effective population size (*N*_e_), as derived using *d*_max_ = 2 *N*_e_  *μ*, varied from 2516 to 4776. The relative strength of selection (*S* = |2 *N*_e_  *s*|) was positively correlated with *S*_e_ (*ρ*_Spearman_ = 0.95, *P* < 2.2 × 10^−16^) (Fig. 4B) as expected but appeared to be negatively correlated with *L*_e_ (*ρ*_Spearman_ = −0.32, *P* = 0.002). Nearly all of the variance of *L*_e_ was explained by the variation in the length of the region under selection (*L*_s_) (*R*^2^ = 0.87) (Fig. 4C). When controlled for this factor, the log ratio of recombination-to-mutation rate (ln r/m) had a significant negative effect (*P* = 0.0011) but the effect of the relative strength of selection (*S*) was insignificant (*P* = 0.84). The slope of ln r/m against *L*_e_ on the standard scale ((x–*μ*)/σ) was only −0.13, compared to that of *L*_s_ (0.97). Thus, while recombination rate is a significant predictor of Effect Length, it is not sufficient to explain the observed range of variation.

A correlation between Effect Length and the efficacy of selection was expected based on our analysis of the stability of mRNA secondary structure towards the 5′-end of genes among real bacterial species. To understand why *L*_e_ and *S* are independent in the simulated data, note that both of these factors are expected to be influenced by the effective population size (*N*_e_), as a high *N*_e_ would increase both the maximum efficacy of selection and the length of the region over which this selection is effective. While multiple linked mutations do reduce effective population size in our simulations as expected (Good et al. 2014), the ratio *N*_e_/*N* is considerably less variable across simulations (Mean: 0.83, SD: 0.09) when compared to over a 2-fold range of variation in both *s* and *L*_s_ (Fig. 4B and C). Thus, the lack of positive correlation between *L*_e_ and *S* of simulated species is presumably due to limited variation in their effective population sizes. In the simulations, the extent of variation in *L*_e_ is predominantly due to the variation in *L*_s_ which, among real species, could be modulated through a combination of effective population size and the actual length of the region under selection. Since *L*_s_ due to selection acting on mRNA secondary structure appears to be limited to a narrow range across species, according to previous works (Molina and Nimwegen 2008) and supported by the analysis in this study (Fig. 3B), alternative explanations may be sought for the extensive purifying selection towards the 5′-end of bacterial genes.

The Spearman correlation between gene length and nucleotide diversity for these 91 simulated species varied over the range of 0.05 to 0.36. It was significantly correlated with the Effect Length, just as for the real species, but showed a considerably lower *R*^2^ (0.17 v/s 0.32) (Fig. 4D). However, this difference was not significant as evaluated by resampling simulated species (*P* = 0.0788, *N* = 63) Two other factors that showed a strong association with the diversity–length correlation were *S*_e_ (*ρ*_Spearman_ = 0.20) and *d*_max_ (*ρ*_Spearman_ = 0.67). Overall, nearly 80% of the variation in the strength of gene-length dependence could be explained by *L*_e_, *S*_e_, & *d*_max_ together (*R*^2^ = 0.78), whereas only 40% of the variation for real species could be explained by these factors (*R*^2^ = 0.39). This makes it clear that purifying selection towards the 5′-end of genes can lead to a correlation between gene length and nucleotide diversity. Yet, almost half of the variance of gene-length dependence for the observed species remains to be understood.

## Discussion

In this study, I sought to identify a basis for a positive correlation between nucleotide diversity and gene length observed previously (Ali and Seshasayee 2020). The pattern of reduced polymorphism toward the TIS explains a considerable proportion of this gene-length dependence. Fitting an ASR model to the observed pattern of mean diversity over sites enabled quantification of the size (*S*_e_) and length (*L*_e_) of this effect across species. With a phylogenetic comparative approach, I found the Effect Length to be correlated with a combination of selective and nonselective factors. Simulations revealed how these evolutionary processes could interact to shape the distribution of Effect Length and thereby, the observed diversity–length correlation.

Effect Lengths of different bacteria showed a positive correlation with their maximal growth rates. Species with smaller doubling times are under stronger selection for optimizing translation rates. As the majority of the mutations will cause a shift away from optimized rates, fast-growing bacteria are expected to incur higher fitness costs, and so such mutants are rapidly eliminated. Alternatively, the strength of selection acting on the starting region of genes could be the same across species but the countering effect of random genetic drift is weaker in fast-growing bacteria. Indeed, Effective population size (*N*_e_) was found to correlate positively with growth rate across bacterial species (Bobay and Ochman 2018). When *N*_e_ is higher, variants that increase the stability of RNA secondary structures around translation start sites can be more effectively removed from the population, resulting in a clear pattern of reduced polymorphism in the starting region of genes, reflected in the higher estimates of *L*_e_. Such patterns of loss of diversity were simulated in this study by varying *s* for a fixed *N*_e_. Varying *N*_e_ instead of *s* will produce similar results since diversity patterns are shaped by the relative strengths of selection and drift (Kimura 1983).

Synonymous codon bias in *E. coli* was found to positively correlate with gene length among genes expressed at comparable levels, and it was argued that it reflected the selection to avoid missense errors in longer proteins due to their higher cost of production (Eyre-Walker 1996a). While this could be true for the set of proteins used in that study, it is expected to lead to a negative correlation between silent-site diversity and gene length on a genome-wide scale, contrary to our observations. The counter-acting selection towards the 5′-end of genes could explain this pattern, such that shorter genes have a lower bias due to conflicting forms of selection, but it was assumed to be relevant for the first 50 bases. The selection to avoid mRNA secondary structure around TIS does indeed appear to be limited to this range (Rocha et al. 1999; Molina and Nimwegen 2008). However, nucleotide diversity of linked neutral sites further downstream in the gene would also be reduced due to this purifying selection (Nordborg et al. 1996). While the variation in recombination rates across bacteria (Vos and Didelot 2009) seems to shape *L*_e_, neither the recombination nor the selection to avoid mRNA secondary structure seems sufficient to explain the observed distribution of Effect Length across species as revealed by evolutionary simulations.

Estimates of synonymous diversity have been used to study mutation rate variation across genes after controlling for many suspected factors (Martincorena et al. 2012). However, gene length was not controlled for in that study and shows a significant correlation with the estimates of synonymous diversity, even after correcting for other correlates (*ρ*_Spearman_ = 0.15, *P* = 1.64 × 10^−14^). Admittedly, there is still substantial unexplained variation in the synonymous diversity of genes. Even for the length dependence of this diversity, as much as 50% of the variance across species remains unexplained. The selection for translational accuracy, as discussed above, may have some contribution to this variance (Eyre-Walker 1996a; Coghlan and Wolfe 2000). The small-scale Hill–Robertson effect, interference among weakly selected mutations linked on the same gene, was proposed to explain a negative correlation between synonymous polymorphism and gene length in *Drosophila* (Comeron et al. 1999). This hypothesis has found support in animals (*C. elegans*, Marais and Duret 2001) and plants (*Populus tremula*, Ingvarsson 2007) and may be relevant in some prokaryotic species with small *N*_e_. Synonymous diversity was also found to be reduced at the 3′-end of genes in *E. coli* due to their overlaps with the following translation initiation region (Eyre-Walker, 1996b). This could be another factor modulating the gene-length dependence of diversity. Much remains to be learned about variation in evolutionary dynamics among proteins, and a variety of mechanisms may be uncovered (Drummond et al. 2006; Li et al. 2012; Park et al. 2013).

It is evident from this study that synonymous sites at the start of a gene are heavily constrained compared to the sites downstream. The loss of diversity, as estimated by the Effect Size *S*_e_ can be anywhere between 30% and 80%, and this would be reflected in the local estimates of *N*_e_ (Gossmann et al. 2011). Given that the extent of this effect varies substantially across species, these regions of reduced polymorphism need to be identified and excluded from the estimation and comparison of effective population size across species. Fitting an ASR model to mean silent-site diversities provides an alternative way of estimating *N*_e_ through the estimate of maximum average diversity *d*_max_ as it presumably reflects the diversity minimally affected by selection.

One final issue with ignoring the nonneutrality of synonymous diversities and their gene-length dependence concerns the detection of genes under positive selection (Kreitman 2000; Bustamante et al. 2005). Common tests of the strength of selection acting on a protein-coding sequence involve the ratio of the rate of nonsynonymous substitutions to that of synonymous substitutions (Charlesworth and Eyre-Walker 2006; Lefébure and Stanhope 2007; Lefébure and Stanhope 2009). Often, the ratio of nonsynonymous to synonymous diversity is used instead of substitution rates (Young et al. 2017; López-Pérez et al. 2020; Moulana et al. 2020), or the method is used for intraspecific comparisons (Chen et al. 2006; Wilson and The CRyPTIC Consortium 2020), both of which can be misleading in their own (Kryazhimskiy and Plotkin 2008; Loo et al. 2020). However, the pattern of reduced variation toward gene starts studied here using synonymous diversity is also visible when synonymous substitution rates are used instead (Eyre-Walker and Bulmer 1993). Therefore, the extent of purifying selection on synonymous sites seen in this study could lead to an overestimation of the prevalence of positive selection (Künstner et al. 2011). Increasing recognition of potentially unknown sources and consequences of even weak synonymous selection continues to advance our ability to identify genuine cases of molecular adaptation (Rahman et al. 2021).

## Materials and Methods

### Selection of Strains

Genome assemblies were acquired from NCBI RefSeq, last accessed on 2020 September 14. From the list of bacterial assemblies present on NCBI, the following were excluded: those missing from RefSeq, those that are miss-classified or uncultured, those without species name, or with the term “Candidatus” in place of a genus name, or “bacterium” in place of a species name. Out of the remaining species in the list, the ones with at least 30 chromosomal or higher level assemblies were selected, giving 75 species with a total of 9230 assemblies. For 24 species with more than 100 assemblies, 100 were randomly selected to reduce the overall computational burden, leaving a final set of 5,272 genomes spread across 75 bacterial species.

For each species, genomes were further filtered to exclude highly similar ones. This was done based on the degree of overlap in their sets of chromosomal nonredundant protein ids. There are two sources of variation among these sets, *viz.*, (i) changes in the amino-acid sequence of homologous proteins, and (ii) changes in the accessory genome. The pairwise dissimilarity between sets was measured with Jaccard distance, i.e. $d_{j}=1-\frac{A\cap B}{A\cup B}$ for protein sets corresponding to genomes A & B. Finally, a set of strains were selected for each species given a pairwise distance matrix such that they differed by at least 1% in their set of protein ids, i.e. *d*_j_ ≥ 0.01. This step brought down the total number of assemblies to 4,939.

### Computing Nucleotide Diversity

Ortholog groups across selected strains of a species were identified using SonicParanoid (v1.3.8) in its fast mode with default parameters (Cosentino and Iwasaki 2019). Single-copy orthologs, present in at least 75% of the analyzed genomes, were selected for further analysis. Amino-acid sequences of orthologs were aligned using Clustal Omega (v1.2.4) (Sievers et al. 2011) and converted to codon alignments using PAL2NAL (v14) (Suyama et al. 2006). Sites with over 70% gaps were excluded. *N*-fold degeneracy of each site was determined using a consensus sequence derived from the alignment with a minimum consensus character threshold of 60%. For each site, nucleotide diversity (π) was calculated as follows:


$$\pi =2\frac{\sum_{ij}\;f_{i}f_{j}}{N(N-1)}$$

where the summation is over all possible pairs of 4 bases and *f*_i_ signifies their counts among *N* sequences.

### Effect Length Estimation

Diversity profiles over sites were quantified with respect to their Effect Size and length i.e. the magnitude and extent of the reduction in mean diversity observed near the start of a gene. Effect Size is defined as the log-2 fold decrease in mean diversity at the start compared to the maximum mean diversity over the first 500 sites. Effect Length is defined as the site at which the mean diversity attains the midpoint of its range. An ASR model (negative exponential) of the following form was used to fit observed mean diversities over 4-fold degenerate sites.


$$\bar{\pi }(l)=d_{max}+(d_{min}-d_{max})e^{-cl}+\varepsilon$$

where $\bar{\pi }(l)$ is the observed mean diversity at a site *l* bases downstream of the translation start site (index 0). *d*_max_ is the maximum mean diversity attainable as *l* goes to +∞, *d*_min_ is the minimum estimated mean diversity at *l* = 0, and *c* governs the relative rate of increase in diversity with distance from gene start. The error (*ε*) in estimating the site-specific mean is assumed to be additive, and independent with variance σ^2^/*w* (Fox and Weisberg 2018). Weights (*w*) are set to the number of genes for each site to take into account fluctuations in the mean due to varying sample sizes. Regression was performed using R’s nonlinear least-squares function nls with its Gauss–Newton algorithm. Initial estimates of model parameters were obtained using R’s SSasymp. Effect Size *S*_e_ and length *L*_e_ were calculated using estimates of *c*, *d*_min_ and *d*_max_, as follows:


$$S_{e}=log_{2}\frac{d_{max}}{d_{min}}$$

$$L_{e}=\frac{ln2}{c}$$

The standard errors of these estimates can be derived from the standard errors of model parameters using the Delta method as follows:


$$\sigma (L_{e})=(ln2)\sigma_{K}e^{-K}\sqrt{\left(1-\sigma_{K}^{2}/4\right)}$$

$$\sigma (S_{e})=\frac{1}{ln2}\sqrt{\left(\frac{\sigma^{2}(d_{max})}{d_{max}^{2}}+\frac{\sigma^{2}(d_{min})}{d_{min}^{2}}-\frac{2\sigma (d_{max},d_{min})}{d_{max}d_{min}}\right)}$$

where σ stands for standard error and *K* = ln *c* is used since SSasymp fits the above model using ln *c*.

### Positional Probabilities for RNA-secondary Structure

Vienna RNA package (v2.4.16) was used to predict RNA secondary structures (Hofacker et al. 1994). The probability of each site remaining unpaired (open) in an RNA secondary structure was computed for every gene in a genome over a region from 100 bp upstream to 200 bp downstream (−100:+200) of the translation start site. Partition functions were used to get the base-pairing probabilities of each site in a thermodynamic ensemble of secondary structures. Probabilities were converted to z-scores following Molina’s method (Molina and Nimwegen 2008), with the flanking regions marked as −90 to −51 and +151 to +190. A z-statistic profile was generated for one reference genome from each species. Effect Length corresponding to selection on mRNA secondary structure *L*_s_ was defined as the first site +5 onwards at which the z-score drops to zero; Effect Size *S*_s_ was measured as the maximum z-score over the same region.

### Estimation of Recombination Rate

Recombination rate for each species was estimated from codon alignments, using the Mcorr program (Lin and Kussell 2019). Alignments of individual genes were first concatenated in a single alignment in XMFA format, which was submitted as input to the program. Mcorr takes a coalescent approach to estimate the mutational (*θ*_p_ = 2 *N*_e_  *μ*) and recombinational divergence (*ϕ*_p_ = 2 *N*_e_  *γ*) of a bacterial population given a sample of genomes, by fitting an analytical model to substitution correlation profile among pairs of synonymous sites. The rate of recombination relative to mutation rate (r/m) was estimated as *ϕ*_p_ /*θ*_p_. Estimates were log-transformed (Box–Cox’s *λ* = 0) for regression analysis.

### Estimation of Codon-usage Bias and Minimum Doubling Times

Codon-usage bias (CUB) was estimated using gRodon package in R (Weissman et al. 2021). gRodon uses CUB of highly expressed genes, and other associated variables, to predict minimum doubling times based on a regression model trained on growth rate data from Vieira-Silva and Rocha 2010, and Madin et al. 2020. For each genome, its multi-FASTA file of coding sequences was used as input to gRodon. First, ribosomal genes were used as the set of highly expressed genes to calculate CUB, which was then used to predict minimum doubling times DT_pred_. Estimates of CUB_HE_ and DT_pred_ were averaged over all genomes for each species. A squared-root transformation was applied to CUB_HE_ (Box–Cox’s *λ* = 0.5) prior to regression analysis, and DT_pred_ was log-transformed (Box–Cox’s *λ* = −0.1).

### Phylogenetic Reconstruction

UBCG program was used to reconstruct bacterial phylogeny based on concatenated amino-acid alignments of 81 universal bacterial markers (Kim et al. 2021). Only one reference genome assembly was used for each bacterial species. In the absence of a reference assembly, as defined in the NCBI RefSeq database, a representative genome was used. For the two species (*Planctomycetes* and *Wolbachia*) that lacked both a reference and a representative genome, the genome with the number of genes closest to the mean was selected. Contigs identified as plasmid by the NCBI annotation pipeline were removed from these reference sequences. Positions with more than 50% gaps were removed from the alignment. A maximum-likelihood phylogeny was estimated using the RAxML program (v8.2.12), with a Jones–Taylor–Thornton (JTT) model of sequence evolution, along with CAT approximation (Stamatakis 2014). UBCG provides branch support in terms of a gene support index (GSI), i.e. the number of gene trees supporting a bipartition in the species tree. The default threshold of a minimum of 95% similarity was used to decide whether a branch is supported by a gene tree. The tree was rooted in between Terrabacteria and Gracilicutes, following a recently published rooted phylogeny of all bacteria (Coleman et al. 2021).

### Phylogenetic Comparative Analysis

A phylogenetic generalized least-squares approach (phylo-GLS) was used to test for relationships among variables across species while accounting for their phylogenetic correlation. The strength of the phylogenetic signal on the distribution of a genetic trait or residuals of a regression model can be quantified using Pagel’s *λ* (Pagel 1999). Pagel’s *λ* is a multiplier of the off-diagonal entries of the phylogenetic covariance matrix wherein a value of 0 signifies independent evolution and a value of 1 signifies the evolution in complete accordance with Brownian motion over shared ancestry (Freckleton et al. 2002). gls function from R package “nlme” was used to perform generalized least squares, and corPagel function from R package “ape” was used to calculate the required correlation structure based on Pagel’s *λ* (Symonds and Blomberg 2014).

### Simulating Selection on Translation Initiation Regions

Forward evolutionary simulations were used to test whether purifying selection on translation initiation regions and recombination are sufficient to explain the correlation between gene length and nucleotide diversity. Simulations were performed using SLiM version 4 (Haller and Messer 2023) following Cury’s extensions to bacteria under the Wright–Fisher model (Cury et al. 2022). A randomly generated 50 Kbp chromosome was used in all simulations. Gene lengths were log-normally distributed, such that the median gene length was approx. 1000 and 95% of the values were in the range of 450 to 2200. The population size was set to 5,000 and the number of generations to 20,000. Other parameters - mutation rate, recombination rate, recombination tract length, strength of selection, the length of the region under selection—were sampled from their observed distributions over the species used in this study. Mutation rates were estimated from the average silent-site diversity using π_S_ ∼ *θ* = 2 *N*_e_  *μ* with *N*_e_ = 5,000. Recombination rates were extracted from Mcorr’s estimates of recombination-to-mutation rate ratios along with the estimates of average recombination fragment lengths. The strength of purifying selection was approximated using the following equation (Equation 3.18, Kimura 1983).


$$\frac{H_{T}}{H_{T,0}}=2\frac{S-1+e^{-S}}{S(1-e^{-S})}$$

where *H*_T_ & *H*_T,0_ was replaced with *d*_min_ & *d*_max_ respectively, and *S* = 2 *N*_e_  *s* was used to get the strength of selection (*s*). The average length of the region of selection was set to the estimated Effect Length. Within the region under selection, all sites had the same selection coefficient but the length of the region was normally distributed over genes. All other sites in the genome were neutral. 100 combinations of parameters were drawn from their observed distributions and 40 replicate simulations were run for each set.

### Statistical Analysis

Variables were transformed, wherever necessary, using Box–Cox transformation to reduce skewness and approximate normality. VIF was used to check and control for strong collinearity among predictors. VIF for a variable is calculated as 1/(1-*R*^2^) of a linear regression in which it appears as a response variable dependent on the rest of the predictors. Regression was performed on scaled variables (using mean and standard deviation) to make coefficients comparable. All statistical analyses were performed using R (v4.1.2).

## Supplementary Material

evae078_Supplementary_Data

## Data Availability

The source codes, plotting scripts, metadata, and analysis outputs of this study can be accessed from the GitHub repository https://github.com/A-Farhan/diversity_length_correlation
