# Supplementary material for: Patterns of Change in Nucleotide Diversity Over Gene Length
Source: Genome Biol Evol. 2024 Apr 12;16(4):evae078. doi: 10.1093/gbe/evae078 (PMC11040516; doi:10.1093/gbe/evae078)
Supplement: evae078_Supplementary_Data [file evae078_supplementary_data.pdf]

SI Text 1: Statistical correlates of L_e

The observed distribution of Effect Lengths across species could be biased by potential confounding variables involved in this analysis, such as the number of strains, number of genes, average gene length, and mean diversity. Additionally, as the frequency of 4-fold degenerate sites is governed by the GC content, such that high GC species will have a higher density of such sites on average within a given coding region, Effect Length could be correlated to GC content as well. Spearman correlation of these 5 variables *viz.*, the number of strains $N_{strains}$, the number of genes N_{genes}, average gene length L_{gene}, average gene diversity $\boldsymbol{\pi}_{gene}$, and GC content P_{GC} with Effect Length was non-significant after multiple-testing correction (Holm-Bonferroni's method), except that for the number of strains and the number of genes [**SI Table 1**]. To be able to control for these correlations in further analyses, and to estimate the proportion of variance in the Effect Length across species explained by these variables alone, a multiple linear regression analysis among these confounders and the Effect Length was performed. First, the distributions of individual variables were analysed for their shapes and the presence of outliers. Nucleotide diversity was log-transformed (Box-Cox $\lambda \approx 0$). *Planctomycetes* bacterium was excluded, on account of its average gene length of 1565 that is 6 standard deviation (SD) greater than the mean of 974 across species. Then, collinearity among predictors was checked by calculating Variance Inflation Factor (VIF) for each predictor variable. VIF for a variable is calculated from a linear regression in which it appears as a response variable dependent on the rest of the predictors. It is calculated as $1/(1-R^2)$ from the corresponding model and is supposed to be high if other predictors can explain a large proportion of the variance in the target variable. GC content had the highest VIF of 2.3, followed by the number of genes (2.1) [**SI Table 1**]. Since GC content also had the weakest Spearman correlation with Effect Length, it was excluded from further analyses. Finally, linear regression of L_e on $N_{strains}$, N_{genes}, L_{gene}, and $\boldsymbol{\pi}_{gene}$ using their scaled distributions (z-scores) revealed that at least one of these factors was significantly associated with the Effect Length (F-statistic = 4.843, P = 0.001911). The linear model explained 20 % of the variation in L_e (R^2 = 0.196) and the greatest effect was due to $N_{strains}$ and N_{genes}.

Effect length appears to be correlated to both the number of strains and the number of genes. However, these analyses assumed independence of observations which may not be true for many genetic traits. The strength of phylogenetic signal in the above model was quantified using Pagel's λ. For a Generalised Least-Squares (GLS) regression of L_e on $N_{strains}$, N_{genes}, L_{gene}, and $\boldsymbol{\pi}_{gene}$, the maximum likelihood estimate of Pagel's λ to be 0.48, significantly greater than 0 (Likelihood ratio test: $\boldsymbol{\Lambda}$ = 5.98, $P_{\chi 2}$(df=1) = 0.014). With this model, N_{genes} was no longer significant [**SI Table 1**]. However, $N_{strains}$ still had a

significant positive effect on L_e (P = 0.004) and accordingly, was controlled for in further analyses.

SI Table 1: Statistical correlates of Effect Length.

	Spearman correlation			Collinearity		Linear Regression		phylo-GLS	
Variables	ρ	P_{raw}	$P_{adjusted}$	R^2	VIF	β	P(|β|>0)	β	P(|β|>0)
$N_{strains}$	0.35	0.004	0.02	-0.010	0.990	0.315	0.009	0.31	0.004
N_{genes}	0.32	0.01	0.042	0.518	2.074	0.290	0.017	0.24	0.094
L_{gene}	-0.29	0.02	0.061	0.397	1.660	-0.093	0.448	-0.03	0.835
π_{gene}	-0.27	0.031	0.061	0.135	1.156	-0.144	0.234	-0.16	0.156
P_{GC}	0.03	0.838	0.838	0.574	2.348	NA	NA	NA	NA

SI Table 2: Growth rate correlates of Effect Length.

	Spearman correlation			Collinearity		Linear Regression		phylo-GLS	
Variables	ρ	P_{raw}	$P_{adjusted}$	R^2	VIF	β	P(|β|>0)	β	P(|β|>0)
N_{rrn}	0.50	2.3×10^{-5}	6.9×10^{-5}	0.39	1.63	0.254	0.0522	0.262	0.0552
CUB-HE	0.53	7.4×10^{-6}	2.9×10^{-5}	0.72	3.62	0.364	0.0061	0.356	0.0082
DT_{pred}	-0.43	4.5×10^{-4}	9.0×10^{-4}	0.71	3.42	NA	NA	NA	NA
$N_{strains}$	0.35	5.0×10^{-3}	5.0×10^{-3}	-0.02	0.98	0.253	0.0142	0.266	0.0088

SI Figure 1: Patterns of nucleotide diversity over 4-fold degenerate sites, for 25 randomly selected *E. coli* genes. Value for each site is an average over the next 120 bases.

SI Figure 2: Maximum-likelihood phylogeny of 75 bacterial species used in this study. The tree was rooted in between Terrabacteria and Gracilicutes. Bootstrap values for a branch represent the number of gene trees out of 81 universal markers supporting the branch in the species tree.

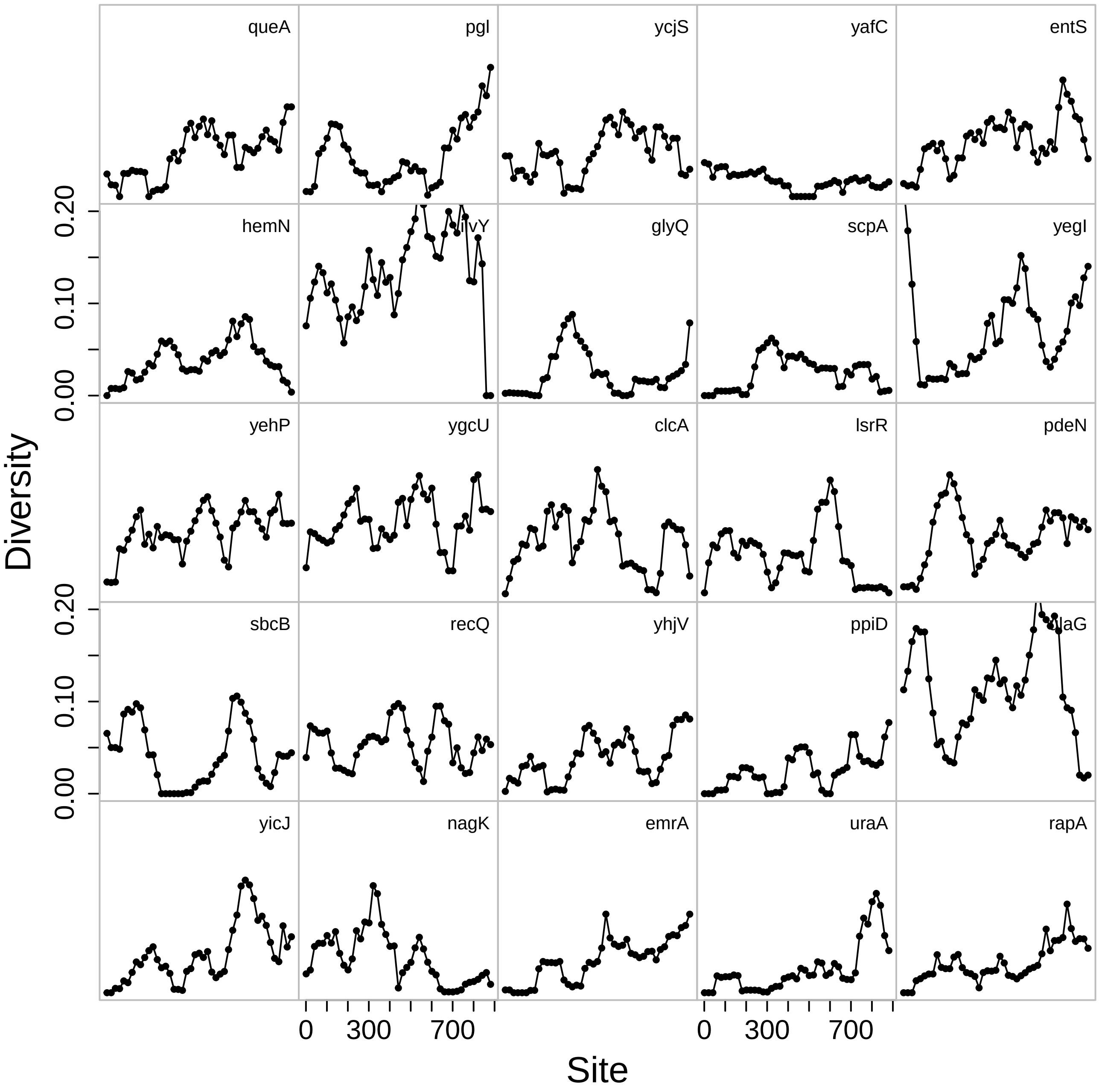
queA
pgl
ycjS
yafC
entS
hemN
ilvY
glyQ
scpA
yegI
yehP
ygcU
clcA
lsrR
pdeN
sbcB
recQ
yhjV
ppiD
dnaG
yicJ
nagK
emrA
uraA
rapA
0.00
0.10
0.20
0
300
700
Diversity
Site

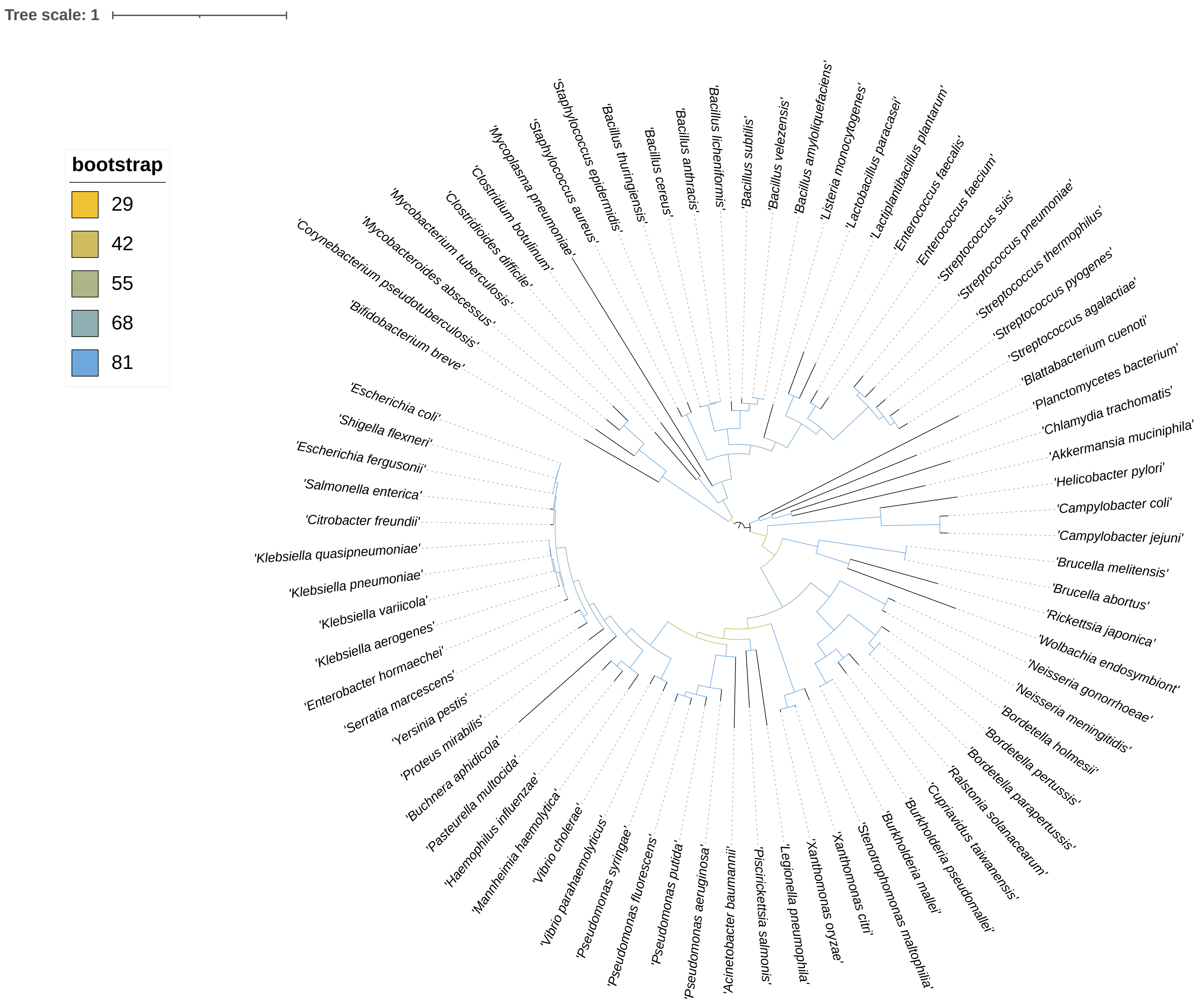

Tree scale: 1
bootstrap
29
42
55
68
81
'Escherichia coli'
'Shigella flexneri'
'Escherichia fergusonii'
'Salmonella enterica'
'Citrobacter freundii'
'Klebsiella quasipneumoniae'
'Klebsiella pneumoniae'
'Klebsiella variicola'
'Klebsiella aerogenes'
'Enterobacter hormaechei'
'Serratia marcescens'
'Yersinia pestis'
'Proteus mirabilis'
'Buchnera aphidicola'
'Pasteurella multocida'
'Haemophilus influenzae'
'Mannheimia haemolytica'
'Vibrio cholerae'
'Vibrio parahaemolyticus'
'Pseudomonas syringae'
'Pseudomonas fluorescens'
'Pseudomonas putida'
'Pseudomonas aeruginosa'
'Acinetobacter baumannii'
'Piscirickettsia salmonis'
'Legionella pneumophila'
'Xanthomonas oryzae'
'Xanthomonas citri'
'Stenotrophomonas maltophilia'
'Burkholderia mallei'
'Burkholderia pseudomallei'
'Cupriavidus taiwanensis'
'Ralstonia solanacearum'
'Bordetella parapertussis'
'Bordetella pertussis'
'Bordetella holmesii'
'Neisseria meningitidis'
'Neisseria gonorrhoeae'
'Wolbachia endosymbiont'
'Rickettsia japonica'
'Brucella abortus'
'Brucella melitensis'
'Campylobacter jejuni'
'Campylobacter coli'
'Helicobacter pylori'
'Akkermansia muciniphila'
'Chlamydia trachomatis'
'Planctomycetes bacterium'
'Blattabacterium cuenoti'
'Streptococcus agalactiae'
'Streptococcus pyogenes'
'Streptococcus thermophilus'
'Streptococcus pneumoniae'
'Streptococcus suis'
'Enterococcus faecium'
'Enterococcus faecalis'
'Lactiplantibacillus plantarum'
'Lactobacillus paracasei'
'Listeria monocytogenes'
'Bacillus amyloliquefaciens'
'Bacillus velezensis'
'Bacillus subtilis'
'Bacillus licheniformis'
'Bacillus anthracis'
'Bacillus cereus'
'Bacillus thuringiensis'
'Staphylococcus epidermidis'
'Staphylococcus aureus'
'Mycoplasma pneumoniae'
'Clostridium botulinum'
'Clostridioides difficile'
'Mycobacterium tuberculosis'
'Mycobacteroides abscessus'
'Corynebacterium pseudotuberculosis'
'Bifidobacterium breve'
